# Estimates of probable dementia prevalence from population-based surveys compared with dementia prevalence estimates based on meta-analyses

**DOI:** 10.1186/1471-2377-10-62

**Published:** 2010-07-21

**Authors:** Kaarin J Anstey, Richard A Burns, Carole L Birrell, David Steel, Kim M Kiely, Mary A Luszcz

**Affiliations:** 1 Centre for Mental Health Research, Australian National University, Canberra, ACT, Australia; 2 Centre for Statistical & Survey Methodology (CSSM), University of Wollongong, Wollongong, NSW, Australia; 3 School of Psychology and Centre for Ageing Studies, Flinders University, Adelaide, SA, Australia

## Abstract

**Background:**

National data on dementia prevalence are not always available, yet it may be possible to obtain estimates from large surveys that include dementia screening instruments. In Australia, many of the dementia prevalence estimates are based on European data collected between 15 and 50 years ago. We derived population-based estimates of probable dementia and possible cognitive impairment in Australian studies using the Mini-Mental State Examination (MMSE), and compared these to estimates of dementia prevalence from meta-analyses of European studies.

**Methods:**

Data sources included a pooled dataset of Australian longitudinal studies (DYNOPTA), and two Australian Bureau of Statistics National Surveys of Mental Health and Wellbeing. National rates of probable dementia (MMSE < 24) and possible cognitive impairment (24-26) were estimated using combined sample weights.

**Results:**

Estimates of probable dementia were higher in surveys than in meta-analyses for ages 65-84, but were similar at ages 85 and older. Surveys used weights to account for sample bias, but no adjustments were made in meta-analyses. Results from DYNOPTA and meta-analyses had a very similar pattern of increase with age. Contrary to trends from some meta-analyses, rates of probable dementia were not higher among women in the Australian surveys. Lower education was associated with higher prevalence of probable dementia. Data from investigator-led longitudinal studies designed to assess cognitive decline appeared more reliable than government health surveys.

**Conclusions:**

This study shows that estimates of probable dementia based on MMSE in studies where cognitive decline and dementia are a focus, are a useful adjunct to clinical studies of dementia prevalence. Such information and may be used to inform projections of dementia prevalence and the concomitant burden of disease.

## Background

In 2005 it was estimated that the number of people with dementia in Australia reached 200,000 [1]. Recent projections indicate that if there is no risk reduction at the population level, the number of people with dementia in Australia will exceed 730,000 by 2050 [2]. In Australia, dementia will cause the largest burden of disease for women and 5th largest for men by 2016 [3]. These Australian figures were derived using a similar methodology to European studies that project future population characteristics by age and sex [4] and took into account prevalence estimates from four meta-analyses of mostly European studies [5-8]. Consequently, estimates of dementia prevalence in Australia are typically based on non-Australian studies and it is necessary to compare those estimates derived from European and North America studies with large scale Australian studies.

Many of the dementia prevalence studies included in the source meta-analyses [5-8] were published 15 to 50 years ago, with the actual data often having been collected years prior to publication. For example, one meta-analysis is based on articles published between 1945 and 1985 [8], another analysed articles published between 1981 and 1991 (mean publication year 1988) [6] and a third covered publications between 1987 and 1994. Not all studies contributing to these meta-analyses were population-based studies as some of the samples were drawn from medical practitioner lists [6]. Diagnostic criteria for dementia have changed since these studies were conducted and the methodology of meta-analyses has also become more rigorous with the publication of guidelines [9,10] and the development of the Cochrane Collaboration.

Whilst acknowledging that meta-analyses are the preferred method for aggregating data on dementia prevalence [1,2], their limitations prompt closer scrutiny of complementary sources of data. This is particularly important because prevalence estimates are used for service planning, and estimates of health care costs and burden of disease. When projected up to the population, small differences in prevalence rates have large implications for estimating health care costs. For example, 1% increase in dementia for the year 2003 in Australia would have cost approximately AUD $217 million [11].

The aim of the present study was to compare prevalence of probable dementia and cognitive impairment using MMSE cut-offs [12] with dementia prevalence estimates[1,2] generated by application of techniques used in meta-analyses of European studies. The MMSE, was chosen because it is the most widely used screening instrument for dementia. Furthermore, the MMSE is used almost universally in clinics [13], is recommended as a dementia screen by the U.S. Preventive Services Task Force [14], has been translated into many languages, and has psychometric properties that have been well documented in many contexts [15].

The current methodology was adopted not only because existing projections have relied on estimates that are at least 15 years old and largely based on meta-analyses of European data, but also because there is no national study of dementia prevalence in Australia that uses clinical diagnoses. This is in part due to the cost of acquiring dementia diagnoses and the logistical difficulty of conducting a national prevalence study over such a large land mass, in comparison to geographically smaller countries. Data come from two sources: Australian Bureau of Statistics (ABS) Surveys and a pooled, harmonized, dataset from DYNOPTA [16]. The latter includes three independent regional studies that have reported dementia prevalence in Australia, but none of these studies individually is large enough on which to base national estimates. These include the Sydney Older Persons' study (SOPS) that used a clinical diagnosis [17,18], the Canberra Longitudinal Study (CLS) that used an algorithm based on data collected by a trained interviewer [19], and the PATH Through Life Study (PATH) [20] that includes a clinical assessment but which to date has identified very few cases because the sample is too young. These three studies all included the Mini-Mental State Examination as did a fourth study contributing to DYNOPTA, the Australian Longitudinal Study of Ageing (ALSA). While the latter did not include a clinical assessment of dementia, it included a cognitive assessment, and cognitive decline and impairment has been a focus of the investigators. The ABS surveys recently have incorporated the MMSE, providing an additional source of data to evaluate for the estimation of probable dementia prevalence.

## Methods

### Data sources

Two types of population-based MMSE data were analyzed to obtain estimates of the prevalence of probable dementia and possible cognitive impairment among older Australians [1,2]. The first type of data was drawn from the *Dynamic Analyses to Optimise Ageing (DYNOPTA) *dataset [16]. DYNOPTA comprises a pooled dataset comprising information from nine Australian Longitudinal Studies of Ageing (N = 50652). Data were harmonised from the contributing studies to create an entirely new and unique dataset. The research program of DYNOPTA focuses on four outcomes that contribute greatly to the burden of disease and disability, namely *dementia and cognition, mental health, sensory impairment, and mobility/activity limitations*. The present study forms part of the program on dementia and cognition. Four studies contributing to DYNOPTA had cognitive decline or dementia as a key focus of investigators and included SOPS, CLS, PATH and ALSA. These studies contributed data on the MMSE among participants aged 65 and older (n = 3908). SOPS used a stratified sampling design, combining a random sample from a Department of Veteran's Affairs listing of veterans and war widows over the age of 75 and a community area random sample of census collection districts from 8 Local Government Areas in the inner west of Sydney during the years 1991-1994. Clinical interview and cognitive assessments were made by a trained geriatrician providing clinical diagnoses of dementia against which to validate the MMSE cutoffs used in the current study. The CLS, PATH and ALSA were all sampled from the electoral roll, and voting is compulsory in Australia. ALSA randomly sampled both community dwelling and institutional residents aged over 70 and also sampled by convenience co-residents and partners aged over 65 years from the Adelaide metropolitan area during the years 1992-1993. CLS randomly sampled both community dwelling and institutional residents aged over 70 from the Australian Capital Territory and Queanbeyan during 1991. CLS used a diagnostic algorithm to generate DSM-III diagnoses of dementia based on a cognitive assessment. These diagnoses were used to evaluate the clinical cutoffs for the MMSE used in DYNOPTA in addition to the data provided by SOPS.

The second type of population-based data was the ABS National Surveys of Mental Health which were conducted in 1997 and 2007. The 1997 National Survey of Mental Health and Wellbeing of Adults (97 NSMH) was a stratified, multistage area sample of private dwellings framed by 1991 census collection districts. The sample included 10641 participants aged over 18, of whom 1788 were aged 65 or older and completed the MMSE [21]. A second survey conducted in 2007 (07 NSMH), was also framed by a stratified, multistage area sample of private dwellings that excluded remote areas. It included 8800 participants aged between 16 and 85 [22], of whom 1905 were aged 65 and over and completed the MMSE [23]. Table 1 shows the sample characteristics of the datasets.

**Table 1:** Sample characteristics of DYNOPTA, the 97 NSMH and 07 NSMH

	**DYNOPTA**	**97NSMH**	**07NSMH**
	
**Total N**	3908	1788	1905
**Gender (n, %)**			
Male	1954 (50.0%)	728 (40.7%)	904 (47.5%)
Female	1954 (50.0%)	1060 (59.3%)	1001 (52.5%)
**Age (n, %)**			
65-69	441 (11.3%)	576 (32.2%)	636 (33.4%)
70-74	931 (23.8%)	499 (27.9%)	468 (24.6%)
75+	2536 (64.9%)	713 (39.9%)	801 (42.0%)
75-79	1099 (28.1%)	-	444 (23.3%)
80-84	797 (20.4%)	-	357 (18.7%)
85-89	458 (11.7%)	-	-
90-94	140 (3.6%)	-	-
95+	42 (1.1%)	-	-
**Education (n, %) **			-
no formal education	14 (0.4%)	-	-
some or all of primary school or secondary school	2172 (55.6%)	-	-
non-tertiary study (i.e. apprenticeship/trade, certificate, undergraduate diploma	1137 (29.1%)	-	-
tertiary study (bachelor degree, post-graduate diploma, higher degree)	321 (8.2%)	-	-
Missing - No Response/Educated Overseas	264 (6.8%)	-	-
**Finished secondary school**			
Yes	-	502 (28.1%)	420 (22.0%)
No	-	1286 (71.9%)	1485 (78.0%)

### Data preparation and variable description

MMSE item and total scores were imputed for DYNOPTA, 97 NSMH and 07 NSMH. For the DYNOPTA dataset, MMSE items from contributing studies were harmonized to provide equivalent scoring. Multiple imputation, using the MICE module in STATA v.10, computed total MMSE scores from available item level data, age, sex, and education with a cohort variable that reflected the contributing study from which a participant was included. Five imputed datasets were computed from which total imputed scores were averaged to create an MMSE total score for the DYNOPTA data file. Due to study differences in coding of non-response, a conservative approach was taken whereby all missing data was imputed, regardless of nature of non-response. Based on validation work of the imputation undertaken by the authors (not reported here), cases with more than 50% missing data (n = 105) on the MMSE were excluded. A further 437 (10.97%) had missing item level data on the MMSE which was imputed. For the NHMS datafiles the rates of missing data at item level were much lower for both the 97 (n = 61; 3.4%) and '07 (n = 88; 4.6%) datasets. The NSMH was imputed using the same method as that described for DYNOPTA. Categories based on MMSE scores were also created and divided according to probable dementia (MMSE<24), possible cognitive impairment (MMSE = 24 to 26) and no cognitive impairment (MMSE >26).

The cutoff of 23/24 for probable dementia is widely recommended and has been validated in studies of the sensitivity and specificity of the MMSE [24-27]. The range from 24 to 26 has less empirical support for defining possible cognitive impairment than the cut-off of < 24 does for defining probable dementia. It was based on the only Australian epidemiological data on MMSE ranges in mild cognitive disorders from the PATH Through Life Study [20]. This study provided clinical diagnoses of mild cognitive disorders that were used to validate the MMSE score.

### Demographic variables

Demographic variables included in the analyses were age-group, sex and education. The 97NMHS only recorded a categorical age value for participants aged over 75, so the data for this age-group is aggregated. The education variable for DYNOPTA included four levels (No formal education, some or all primary or secondary school, post-secondary, and tertiary study). The education variable for the 97 NSMH was a binary variable indicating whether the respondent had finished secondary school. A compatible binary variable was created from the 07 NSMH item asking the highest year of school completed.

### Study weights and Statistical analyses

To produce estimates from a sample survey weights are usually calculated to account for differences in probabilities of selection and to ensure consistency with population benchmarks. Weights are provided for NSMH participants at both time waves. For the DYNOPTA population, weights were already available for ALSA but CLS, PATH and SOPS did not have study weights available. For each of these studies weights were developed to adjust the sample in each study to the specific population from which it was drawn and account for the probability of selection of each individual. These weights were then benchmarked to the Estimated Resident Population (ERP) for the relevant year, sex, age-group and geographical area. For ALSA, the 1992 ERP for Adelaide was used; for CLS, the 1991 ERP for ACT was used; for PATH, the 2001 ERP for ACT was used; and for SOPS, the 1991 ERP for the two relevant groupings of Local Government Areas (LGA) in Sydney were used. To enable analysis using the pooled data set, which consists of the data from the four studies, final combined weights were calculated. The final weights combined the study specific weights according to their contributing sample sizes to the pooled data set. These combined weights were then benchmarked by age-group and sex to the 1991 Australian ERP and then used in the estimation. Although the PATH study was conducted in 2001, it only contributed to the 65-69 year age-group. The estimated standard errors for the prevalence estimates take into account the use of weights and the complex survey design for each study and also the number of multiple imputations used in the analysis. Using the combined weights in the estimation procedure adjusts for the unequal probabilities of selection in the studies and allows for the contribution of the individual study to the pooled data set. For the mean estimates reported in Table 2, the weighted estimates were very similar to the unweighted estimates. For the estimation of the proportions in Table 3, the weights had more impact on the proportions when the sample size within the age-group by sex cell is small, such as in the over 90 age-groups for males.

**Table 2:** Mean (standard error) of MMSE scores for the DYNOPTA baseline population and NSMH (aged 65+) as a whole, by gender and by age cohort

		DYNOPTA				97NSMH				07NSMH			
**Age Groups**		**N**	**Mean**	**Std. Err**	**95% Conf. Interval**	**N**	**Mean**	**Std. Err**	**95% Conf. Interval**	**N**	**Mean**	**Std. Err**	**95% Conf. Interval**

65-69	Total	441	28.73	0.13	28.48 - 28.98	576	27.71***	0.14	27.09 - 28.34	636	28.70	0.08	28.49 - 28.90
	Males	162	28.69	0.17	28.35 - 29.04	283	27.28**	0.18	26.37 - 28.20	333	28.62	0.10	28.28 - 28.96
	Females	279	28.77	0.20	28.38 - 29.16	293	28.16	0.17	27.68 - 28.63	303	28.76	0.11	28.50 - 29.03
													
70-74	Total	931	27.58	0.12	27.34 - 27.82	499	27.20	0.16	26.49 - 27.92	468	28.39*	0.10	28.14 - 28.64
	Males	483	27.43	0.12	27.19 - 27.67	200	26.80*	0.28	25.93 - 27.66	241	28.30	0.14	28.01 - 28.60
	Females	448	27.71	0.18	27.35 - 28.07	299	27.49	0.19	26.75 - 28.22	227	28.47	0.14	28.14 - 28.80
													
75+	Total	2536	26.27	0.09	26.08 - 26.45	713	26.40	0.17	24.94 - 27.87	801	27.90**	0.09	27.69 - 28.11
	Males	1309	26.35	0.11	26.14 - 26.56	245	26.36	0.33	24.41 - 28.30	330	27.75	0.14	27.36 - 28.15
	Females	1227	26.22	0.13	25.97 - 26.47	468	26.43	0.19	25.14 - 27.71	471	28.02	0.11	27.78 - 28.26
													
75-79	Total	1099	26.98	0.11	26.76 - 27.21					444	28.07**	0.12	27.97 - 28.16
	Males	589	26.95	0.14	26.68 - 27.22					198	27.82*	0.19	27.67 - 27.97
	Females	510	27.01	0.16	26.69 - 27.33					246	28.26	0.15	28.15 - 28.38
													
80-84	Total	797	26.06	0.17	25.73 - 26.39					357	27.77**	0.13	27.67 - 27.87
	Males	412	26.04	0.20	25.64 - 26.44					132	27.86	0.19	27.71 - 28.02
	Females	385	26.07	0.24	25.61 - 26.54					225	27.72	0.17	27.58 - 27.85
													
85-89	Total	458	25.35	0.21	24.94 - 25.76								
	Males	246	25.19	0.27	24.66 - 25.73								
	Females	212	24.43	0.28	24.88 - 25.98								
													
90-94	Total	140	24.26	0.41	23.46 - 25.06								
	Males	52	23.35	0.84	21.69 - 25.00								
	Females	88	24.53	0.46	23.63 - 25.42								
													
90+	Total	182	23.48	0.39	22.71 - 24.24								
	Males	62	23.15	0.75	21.67 - 24.63								
	Females	120	23.56	0.45	22.68 - 24.43								
													
95+	Total	42	21.20	0.87	19.50 - 22.91								
	Males	10	22.03	1.84	18.36 - 25.69								
	Females	32	21.10	0.94	19.25 - 22.95								

* p < .05; ** p < .001 t-test analyses between gender were computed within each study for each Age Group category. t-test analyses for between DYNOPTA and both NSMH surveys were computed for total within each Age Group category.

**Table 3:** Proportion of samples with probable dementia and possible cognitive impairment in DYNOPTA and by by gender and by age cohort

		DYNOPTA			97NSMH			07NSMH		
		Male	Female	Total	Male	Female	Total	Male	Female	Total
**Age**	**Dementia Category**	**Proportion** **(95% CI)**	**Proportion** **(95% CI)**	**Proportion** **(95% CI)**	**Proportion** **(95% CI)**	**Proportion** **(95% CI)**	**Proportion** **(95% CI)**	**Proportion** **(95% CI)**	**Proportion** **(95% CI)**	**Proportion** **(95% CI)**

65-69	Probable dementia	3.02 (0 - 7.20)	4.47 (0.55 - 8.39)	3.78 (0.87 - 6.69)	6.72 (2.27 - 11.18)*	5.70 (2.58 - 8.83)	6.22 (3.13 - 9.32)***	04.63 (0.67 - 8.59)	3.43 (0.02 - 6.83)	4.00 (1.51 - 6. 50)*
	Possible cognitive impairment	5.92 (1.33 - 10.51)	3.74 (0 - 7.92)	4.78 (1.63 - 7.93)	18.65 (13.53 - 23.78)	11.07 (7.32 - 14.82)	14.91 (11.69 - 18.13)	5.72 (2.85 - 8.59)	6.35 (3.43 - 9.28)	6.05 (4.00 - 8.11)
	Not Impaired	91.06 (85.77 - 96.35)	91.79 (86.37 - 97.21)	91.44 (87.60 - 95.28)	74.62 (68.15 - 81.08)	83.23 (78.50 - 87.95)	78.86 (74.56 - 83.17)	89.65 (84.71 - 94.59)	90.22 (86.36 - 94.08)	89.95 (87.03 - 92.86)

70-74	Probable dementia	6.22 (3.14 - 9.30)	4.30 (0.75 - 7.84)	5.16 (2.57 - 7.74)	11.16 (5.55 - 16.77) *	7.66 (3.66 - 11.66)	9.09 (5.42 - 12.77)*	4.34 (1.39 - 7.29)	5.70 (2.05 - 9.36)	5.02 (2.47 - 7.58)***
	Possible cognitive impairment	18.95 (15.16 - 22.75)	16.22 (11.16 - 21.28)	17.44 (14.29 - 20.59)	21.13 (14.39 - 27.87)	13.53 (9.45 - 17.61)	16.64 (12.94 - 20.35)	11.66 (6.42 - 16.90)	7.07 (3.20 - 10.95)	9.35 (6.38 - 12.32)
	Not Impaired	74.83 (70.57 - 79.09)	79.49 (74.77 - 84.20)	77.40 (74.23 - 80.57)	67.71 (59.72 - 75.69)	78.82 (73.37 - 84.26)	74.26 (69.34 - 79.18)	84.00 (78.77 - 89.23)	87.23 (82.32 - 92.13)	85.63 (82.16 - 89.14)

75+	Probable dementia	15.33 (12.90 - 17.76)	16.15 (13.70 - 18.59)	15.84 (14.03 - 17.65)	8.70 (0.99 - 16.41) *	14.69 (8.54 - 20.83)	12.63 (6.41 - 18.85)	8.51 (4.61 - 12.42)	4.91 (2.91 - 6.92)	6.55 (4.50 - 8.59)**
	Possible cognitive impairment	24.75 (21.01 - 28.49)	23.25 (20.04 - 26.46)	23.81 (21.42 - 26.20)	29.01 (22.49 - 35.53)	22.11 (17.75 - 26.48)	24.48 (20.83 - 28.13)	11.14 (7.63 - 14.64)	14.29 (9.63 - 18.95)	12.86 (9.96 - 15.75)
	Not Impaired	59.92 (56.24 - 63.59)	60.60 (56.95 - 64.25)	60.35 (57.63 - 63.06)	62.29 (52.94 - 71.64)	63.20 (56.14 - 70.26)	62.89 (56.08 - 69.69)	80.35 (75.02 - 85.68)	80.80 (76.08 - 85.52)	80.59 (77.10 - 84.09)

75-79	Probable dementia	10.74 (7.61 - 13.87)	10.55 (7.17 - 13.93)	10.63 (8.27 - 12.99)	-	-	-	11.26 (4.97 - 17.56)*	4.20 (1.39 - 7.01)	7.53 (4.07 - 10.99)***
	Possible cognitive impairment	21.04 (16.44 - 25.64)	19.00 (14.75 - 23.26)	19.85 (16.75 - 22.94)	-	-	-	9.12 (4.52 - 13.72)	11.37 (5.53 - 17.20)	10.31 (6.24 - 14.38)
	Not Impaired	68.22 (63.33 - 73.11)	70.45 (65.47 - 75.42)	69.52 (65.92 - 73.13)	-	-	-	79.61 (4.52 - 13.72)	84.43 (78.41 - 90.46)	82.16 (77.20 - 87.13)

80-84	Probable dementia	16.92 (12.40 - 21.44)	15.97 (11.44 - 20.49)	16.32 (12.99 - 19.64)	-	-	-	4.55 (0.86 - 8.24)	5.79 (2.79 - 8.79)	5.26 (3.06 - 7.45)***
	Possible cognitive impairment	28.47 (20.15 - 36.78)	24.23 (18.96 - 29.51)	25.79 (21.33 - 30.25)	-	-	-	14.04 (6.97 - 21.11)	17.87 (10.87 - 24.87)	16.22 (11.61 - 20.83)
	Not Impaired	54.61 (47.14 - 62.09)	59.80 (53.83 - 65.78)	57.90 (53.22 - 62.58)	-	-	-	81.41 (74.37 - 88.45)	76.34 (69.48 - 83.20)	78.53 (73.75 - 83.30)

85-89	Probable dementia	25.13 (17.82 - 32.44)	21.02 (14.37 - 27.67)	22.36 (17.49- 27.24)	-	-	-			
	Possible cognitive impairment	31.71 (21.56 - 41.86)	31.27 (22.14 - 40.39)	31.41 (24.34 - 38.49)	-	-	-			
	Not Impaired	43.16 (34.59 - 51.73)	47.71 (38.71 - 56.71)	46.23 (39.00 - 53.45)	-	-	-			

90-94	Probable dementia	41.27 (23.71 - 58.83)	29.89 (19.25 - 40.52)	32.43 (23.40 - 41.46)						
	Possible cognitive impairment	26.95 (13.61 - 40.29)	35.30 (23.74 - 46.87)	33.43 (23.91 - 42.96)						
	Not Impaired	31.78 (17.42 - 46.14)	34.81 (23.60 - 46.01)	34.13 (24.83 - 43.43)						

90+	Probable dementia	42.96 (26.88 - 59.05)	41.03 (31.27 - 50.80)	41.41 (33.08 - 49.74)	-	-	-			
	Possible cognitive impairment	28.20 (16.37 - 40.03)	29.48 (19.92 - 39.04)	29.23 (21.19 - 37.27)	-	-	-			
	Not Impaired	28.84 (15.97 - 41.70)	29.48 (20.22 - 38.75)	29.36 (21.52 - 37.20)	-	-	-			

95+	Probable dementia	52.75 (10.75 - 94.75)	69.39 (52.56 - 86.23)	67.52 (51.69 - 83.36)						
	Possible cognitive impairment	35.46 (0 - 75.17)	14.67 (0.93 - 28.41)	17.01 (4.59 - 29.43)						
	Not Impaired	11.79 (0 - 33.86)	15.94 (1.23 - 30.65)	15.47 (2.55 - 28.40)						

* p < .05; ** p < .01; *** p < .001 Chi-Square analyses between gender and MMSE category reported in Male column. Chi-Square analyses between Study and MMSE category reported in Total column under the NMHS surveys (ref is DYNOPTA). Yates' correction used where appropriate

Comparisons between datasets were conducted using tests of proportions. Logistic regression was used to evaluate gender by age interactions in prevalence of probable dementia. The sensitivity and specificity of the newly created MMSE probable dementia diagnosis in DYNOPTA was validated by comparing it with data available on clinical diagnoses for the SOPS [17] and CLS [19] studies that contributed to the DYNOPTA pooled dataset. Sample weights were used in analyses of DYNOPTA, the 97 NSMH and the 07 NSMH.

## Results

### Description of samples

The sample characteristics of the DYNOPTA, 97 NSMH and 07 NSMH are shown in Table 1. DYNOPTA has equal proportions of males and females, while the 97 NSMH (χ^2 ^= 61.447; df = 1; p < .001) and 07 NSMH (χ^2 ^= 4,738; df = 1; p < .05) have higher proportion of females. The 97 NSMH lacks information on age by year for adults aged over 75 years which limits comparison with other data.

The information on education for each study was different and hence could not be harmonized. In DYNOPTA, whilst nearly half the sample completed some or all schooling, 37.3% reported post-secondary training, including 8.2% who undertook some tertiary studies. In the 97 NSMH and 07 NSMH only 28% and 21% reported finishing their secondary schooling respectively.

### Sensitivity and specificity of the MMSE in DYNOPTA

The CLS clinical diagnosis was based on two algorithms based on the DSM-IIIR [28]or ICD-10 criteria [29]. For the CLS, the MMSE specificity for 23/24 cutoff for diagnosing probable dementia, was 96% (CI 92%-99%) and sensitivity was 75% (CI = 73% to 75%). The SOPS clinical diagnoses were conducted by clinicians using DSM-IIIR [28], DSM-IV [30], and NINCDS [31] criteria. Using the clinical diagnoses of dementia from the SOPS study, the 23/24 threshold on the MMSE had a 91% (CI = 88%-94%) sensitivity and 60% (CI = 58%-62%) specificity. Using data from both studies the 23/24 MMSE cutoff for probable dementia had a 93.06% (CI: 90.66-94.89%) sensitivity and 70.20% (CI: 69.09% to 71.28%) specificity. Clinical data were not available to evaluate the validity of the MMSE cut-off in the 97 NSMH or 07 NSMH.

### Comparison of mean MMSE scores by age and sex

Table 2 shows the mean MMSE scores by age groups and sex for all studies. As the breakdown of ages over 75 was not available for the 97 NSMH, and cognitive impairment increases with age, it was not possible to determine whether differences in mean MMSE scores above 75 were due to different age distributions between studies. Similarly the 07 NSMH only included data up to age 84 so that beyond this age, there are no available survey data within Australia for comparison with DYNOPTA.

With few exceptions, results across datasets were highly similar. Comparison of the 97 NSMH survey with DYNOPTA showed that the mean total MMSE was lower in the 97 NSMH than in DYNOPTA for males aged 65 to 69, and 70 to 74. However, comparison of the 07 NSMH survey and DYNOPTA showed that the total score was lower in DYNOPTA for ages 70-74, 75+ and 75-79, and 80-84.

The 07 NSMH had higher mean MMSE scores than the 97 NSMH in the 65-69 (t(1338) = 6.73, p < .001) 70-74 (t (1.58) = 6.59, p < .001) and 75+ age-groups (t (1672) = 8.71, p < .001).

### Comparison of probable dementia and possible cognitive impairment by age and sex

Table 3 shows the proportion of the DYNOPTA and NSMH samples with probable dementia and possible cognitive impairment according to the MMSE, split by sex and age-group. There were no significant sex differences in rates of probable dementia or impairment in the DYNOPTA sample. Probable dementia was more prevalent in males than females in the 65-69 age-group and 70-74 age-group of the 97 NSMH. The finding that probable dementia was less prevalent in males than females in the lower rates in the 75+ age-group is difficult to interpret because information was unavailable on the distribution of males and females in this age-group.

When males and females were combined to give a total prevalence rate, the 97 NSMH sample for 65-69 and the 70-74 year age groups had higher rates of probable dementia than DYNOPTA. When males and females were combined to give a total prevalence rate for the 07 NSMH sample, there was a higher rate of probable dementia in the 65-69 age-group, but lower rates in the 70-74 and 75+ age-groups in comparison with DYNOPTA. There were no significant differences between the 97 NSMH and 07 NSMH in rates of probable dementia.

### Comparison of MMSE derived cut-offs with prevalence of dementia from meta-analyses

**Figure 1:**
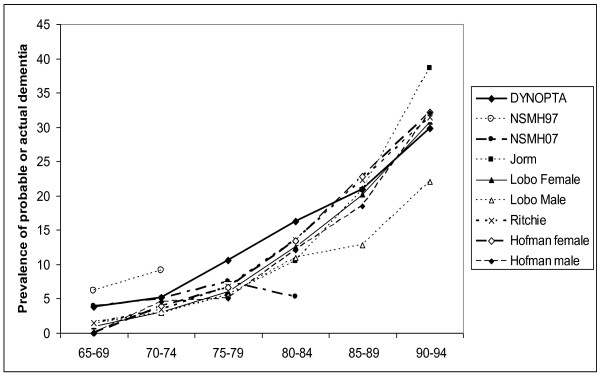
**Comparisons of age trajectories of probable dementia from Australian Surveys and meta-analyses of European Studies**.

Table 4 shows the rates of probable dementia from DYNOPTA and the NSMH surveys compared with the prevalence of dementia from four meta-analyses included in the article by Jorm et al. [2]. For those aged 65-69, and 70-74 the rates of probable dementia from MMSE estimates are clearly higher than the estimates of dementia prevalence from clinical diagnoses, and the 97 NSMH stands out far above all other data sources. For the 75-79 and 80-84 age-groups DYNOPTA estimates are higher than the clinical estimates. For the 85-89 age-group and 90-94, the estimates from DYNOPTA are very similar to estimates from studies of clinical dementia. Estimates above the age of 95 are difficult to evaluate due to small numbers. The estimate from 07 NSMH for the 80-84 age-group appears to be far lower than any other published estimate. Figure 1 shows similar trajectories with age, for the prevalence of probable dementia derived from DYNOPTA and the meta-analyses. If the DYNOPTA estimates were projected onto the Australian population in 2008, the prevalence of dementia in the 65-69, 70-74, 75-79, 80-84 and 85+ age-groups would be 31450, 34232, 58479, 69173 and 148344 respectively [32].

**Table 4:** Prevalence rates of probable dementia from Australian Surveys and prevalence rates of dementia from meta-analyses of European Studies

	MMSE			Clinical Diagnoses					
		
Age Groups	**DYNOPTA **[1]	**NSMH97 **[21]	**NSMH07 **[22]	**Jorm **[2]	**Lobo **[7]**Female**	**Lobo **[7]**Male**	**Ritchie **[5]	**Hofman **[6]**Female**	**Hofman **[6]**Male**
60-64	-			0.7	-	-	-	-	-
65-69	3.78	6.22	4	1.4	1	1.6	1.5	-	-
70-74	5.16	9.09	5.02	2.8	3.1	2.9	3.5	3.9	4.6
75-79	10.63	-	7.53	5.6	6	5.6	6.8	6.7	5
80-84	16.32	-	5.26	10.5	12.6	11	13.6	13.5	12.1
85-89	22.36	-	-	20.8	20.2	12.8	22.3	22.8	18.5
90-94	32.43	-	-	38.6	30.8	22.1	31.5	32.2	32.1
90+	41.41	-	-	-	-	-	-	-	-
95+	67.42	-	-	-	-	-	44.5	36	31.6

### Education and MMSE score

In DYNOPTA, MMSE scores differed according to level of educational attainment. Pair-wise contrasts revealed that individuals with tertiary qualifications (mean = 28.86, SEM = 0.99, n = 321) scored higher than those with post-secondary qualifications (mean = 27.93, SEM = 0.83, n = 1137, p < 0.001), secondary schooling only (mean = 27.02, SEM = 0.86, n = 2172, p < 0.001) or no formal education (mean = 23.66, SEM = 1.19, n = 14, p < 0.001). In both the 97 NSMH and the 07 NSMH, those reporting finishing secondary school had higher MMSE scores than those reporting not finishing secondary school (i.e. 27.48 vs 26.83 (t ( 1786 ) = 3.212, p = .001 and 28.79 vs 28.10 (t(1903 ) = 6.770, p = .000), respectively).

## Discussion

The present study reports prevalence rates of probable dementia and possible cognitive impairment from the two largest sources of population-based data available in Australia, and compares these with published estimates of dementia prevalence based on meta-analyses of dementia prevalence from European studies. Overall the prevalence of probable dementia derived from the DYNOPTA dataset were comparable to estimates derived from meta-analyses. The NSMH surveys showed less consistency with the meta-analyses. The differing pattern of results from these surveys and the limited availability of age data in them suggest that these are not suitable sources of information for making projections about dementia or cognitive impairment in Australia. A possible explanation for the greater congruence of DYNOPTA with the meta-analyses is that DYNOPTA MMSE data were obtained from investigator-led epidemiological studies where dementia and cognitive decline were a key focus. This may have led to higher quality training of assessors, and hence more reliable data collection and coding.

The sensitivity and specificity of the MMSE cutoff used in the DYNOPTA data was highly acceptable. Evaluation of our data showed that the MMSE has a high false positive rate, suggesting that the figures for DYNOPTA may overestimate the true level of probable dementia. A high false positive rate is a characteristic of all screening instruments used to detect low prevalence disorders, and is not a limitation of the MMSE per se [33]. It therefore appears that the DYNOPTA estimates are a reasonable guide to the levels of cognitive impairment in Australia but may slightly overestimate rates of actual dementia. The prevalence of dementia in Australia based on the 2008 population statistics [32] are likely to represent the upper end of probable dementia prevalence.

In the 97 NSMH men had higher rates of probable dementia than women in the 65-69 and 70-74 age-groups. In the DYNOPTA sample, there were no significant differences in rates of probable dementia between men and women. It is possible that this difference between the survey data and some of the meta-analyses that found higher rates of dementia in women is due to sampling biases in the studies that contributed to the meta-analyses. Population weights were applied to all the survey datasets to account for sampling bias, whereas this was not done for the studies contributing to meta-analyses, or in the meta-analyses themselves. Alternatively, the higher life expectancy of Australian men compared to that of males in a number of European countries [34] may influence the population level of cognitive impairment among men. Other possible explanations for the difference in prevalence patterns by gender include differences in sampling frames between studies, different risk factor profiles between countries, and possibly the higher of education among women in Australia due to the more recent data collection. Given the importance of establishing gender differences in prevalence for developing projections of need for dementia care, there is a need to further investigate gender differences in studies with clinical diagnoses.

All data sources have limitations. Prevalence studies used in meta-analyses may have sample bias, with participants who complete clinical assessments having higher socio-economic status and education than the general population. They often under-represent minority groups, individuals with low literacy, and those living in rural or remote locations. Similarly, DYNOPTA and NSMH surveys under-represented minority groups including indigenous Australians, those from culturally and linguistically diverse groups and rural and remote locations. None of the data reports on dementia prevalence have systematically evaluated possible cohort effects. With the increase in IQ scores observed over the past several decades (known as the Flynn Effect) [35] and the general increase in health amongst cohorts moving into old age, it is possible that a reduction in dementia prevalence at older ages will be observed due to greater cognitive reserve [36].

The MMSE has several limitations as a means of categorizing individuals with dementia and these are well described in the literature [24,37,38]. Scores on the MMSE are related to level of education, although education only accounts for a small proportion of variance in scores [39]. The MMSE appears to have unacceptably high misclassification rates among adults who are illiterate [40] but has been shown to be reliable for use in the oldest old [41]. A strength of our study is the reporting of standard errors and the use of population weights, whereas confidence intervals of estimates from meta-analyses were not reported. Hence, the degree of error associated with the estimates from meta-analyses is unknown.

## Conclusions

The data reported here confirm recent projections of the numbers of persons with dementia in Australia [1] and indeed suggest they may even be conservative. An overview of all the results from available sources suggests that the rate of dementia roughly doubles every 5 years between the ages of 70 and 84, but that the rate of increase slows thereafter. We conclude that MMSE data from population-based studies for which dementia or cognitive decline is a focus, provide a valuable adjunct to information on dementia prevalence derived from meta-analyses.

## Competing interests

The authors declare that they have no competing interests.

## Authors' contributions

KJA developed the idea for the manuscript, drafted the manuscript, and interpreted the statistical analyses. RAB conducted some statistical analyses, interpreted the results and contributed to drafting the manuscript. CLB developed the sample weights, conducted some of the statistical analyses, and contributed to drafting the manuscript. DS developed the sample weights, conducted some of the statistical analyses, and contributed to drafting the manuscript. KMK conducted some statistical analyses, interpreted the results and contributed to drafting the manuscript. MAL contributed to interpretation of results and drafting of the manuscript. All authors read and approved the final manuscript.

## Pre-publication history

The pre-publication history for this paper can be accessed here:

http://www.biomedcentral.com/1471-2377/10/62/prepub
